# Comparison of chemo-mechanical and conventional caries removal methods in 6-to-12-year-old children: Randomized clinical trial

**DOI:** 10.1371/journal.pone.0339771

**Published:** 2026-01-16

**Authors:** Abdulrahman Bala Malami, Yewande Isabella Adeyemo, Aliyu Aminu, Folakemi Adenike Oredugba

**Affiliations:** 1 Department of Child Dental Health, Bayero University, Kano, Kano State, Nigeria; 2 Department of Child Dental Health, Aminu Kano Teaching Hospital, Kano, Kano State, Nigeria; 3 Department of Medical Microbiology, Bayero University, Kano, Kano State, Nigeria; 4 Department of Medical Microbiology, Aminu Kano Teaching Hospital, Kano, Kano State, Nigeria; 5 Department of Child Dental Health, Lagos University Teaching Hospital, Lagos, Lagos State, Nigeria; Indiana University School of Dentistry, UNITED STATES OF AMERICA

## Abstract

**Background:**

To address the discomfort associated with conventional drilling for caries removal in children, a minimally invasive approach such as chemo-mechanical caries removal was developed. This study aimed to compare the effectiveness of, and patient behavior associated with chemo-mechanical caries removal versus conventional drilling in children aged 6–12 years.

**Methods:**

A split-mouth randomized clinical trial was conducted involving 66 molar teeth. The effectiveness of Papacarie Duo Gel and conventional drilling methods was assessed using colony-forming unit (CFU) counts before and after each treatment. Cavity preparation time and intraoperative patient behavior using the Frankl Behavior Rating Scale were also evaluated.

**Results:**

Both methods resulted in a significant reduction in bacterial counts post-treatment. However, conventional drilling showed a greater percentage reduction in bacterial load (82.9 ± 13.9; t = −4.42, *p* < 0.001) compared to the chemo-mechanical method (68.6 ± 16.6). The mean caries removal time was 692.4 ± 245.9 sec with the chemo-mechanical method, compared to 266.9 ± 101.6 sec with conventional drilling. In terms of behavior, 57.6% of the children exhibited a positive response during chemo-mechanical treatment whereas negative behavior was more commonly observed with conventional drilling.

**Conclusion:**

While conventional drilling remains the better-established and time-efficient method of caries removal, the chemo-mechanical technique serves as a valuable alternative for managing children, reducing anxiety and promoting greater comfort and cooperation during treatment.

**Trial registration:**

PanAfrican Clinical Trial Registry PACTR202310877678533

## Introduction

Dental caries is a major public health burden in most countries and remains the leading cause of tooth loss among children [1]. Teeth with deep carious lesions extending into the dentine are managed using various techniques, one of which is the conventional drilling method using rotary instruments [2]. This technique typically removes both bacterially contaminated and demineralized dentine before restoring the tooth with a suitable restorative material [2]. However, this method often requires the administration of local anaesthesia and is associated with unpleasant sensations such as noise, vibration, and heat generation [3]. These factors contribute to heightened anxiety and reduced cooperation in paediatric patients. Consequently, these notable drawbacks have prompted the search for alternative, more child-friendly methods of caries removal [4]. One such alternative is chemo-mechanical caries removal (CMCR) which is considered less technique-sensitive and promotes the preservation of healthy dental tissues [5]. Advocates of this approach suggest that it often does not require local anaesthesia, making it more comfortable for children especially those who are anxious, young, or uncooperative [6]. Considering the clinical effectiveness of chemo-mechanical methods, other researchers noted that it holds great promise for future dental practice, as it is atraumatic, preserves healthy tooth tissue, and helps to allay fear and anxiety in children [7].

Time efficiency is another important consideration when choosing caries removal method. Many of the newer techniques are less commonly used today, possibly due to the longer time required for their application [8]. As a result, opinions vary on which technique most effectively reduces bacterial load while also being patient-friendly and minimally invasive.

Almost all the literature reporting the efficacy of CMCR versus conventional methods are not from Africa. If proven effective, this will not only improve patient care delivery but also reduce the number of untreated caries in children, as it does not require local anaesthesia.

This study aimed to assess and compare the clinical effectiveness, efficiency of caries removal, and patient behaviour associated with the use of Papacarie duo gel (a chemo-mechanical agent) versus the conventional rotary instrument method in primary and permanent molars of children aged 6–12 years in a paediatric dental clinic.

## Materials and methods

The research was conducted at the Department of Child Dental Health and the Department of Medical Microbiology, at Aminu Kano Teaching Hospital. It was designed as a split-mouth randomized clinical trial which was registered. Each participant received both the chemo-mechanical method (experimental group) and the conventional method (control group) of caries removal. The study population consisted of children aged 6–12 years who met the eligibility criteria and attended the clinic during the study period (between February 6^th^ and May 29^th^ 2022), during which they were recruited and treated. They were followed up until 30^th^ April 2024. Informed consent forms were signed by parents/caregivers of the participants while assent was obtained from children who were 7 years and older.

Inclusion criteria:

- Children between the ages of 6 and 12 years.
- Presence of at least two carious teeth (primary or permanent molars) with an ICDAS score of 4 or 5.
- Carious lesions limited to Class I dentine caries.

Exclusion criteria:

- Teeth with clinical signs of pulpal or periapical pathology, mobility, or deemed unrestorable.
- Children with systemic medical conditions, chronic illnesses, or those on long-term medication.
- Teeth with developmental anomalies, recurrent caries, or failed restorations.
- Highly uncooperative children.

The sample size was calculated using the formula for comparative studies [9], with a mean difference of 8,000 and an assumed effect size of 0.086, derived from a similar previous study [10].


$$\text{n}=[{\left({\text{Z}}_{\alpha /\text{2}}+{\text{Z}}_{\beta }\right)}^{\text{2}}\;\text{x}\;\{\text{2}{\left(\sigma \text{1}-\sigma 2\right)}^{\text{2}}\}/{\left(\mu_{\text{1}}-\mu_{\text{2}}\right)}^{\text{2}}\;$$

A minimum of 30 teeth was required per arm. Accounting for a 10% attrition rate (N = N/1 – r), this yielded a minimum sample size of 33 teeth for each treatment group, giving a total of 66 teeth (33 children) in the study.

Participants with carious lesions scoring ICDAS 4 (underlying dark shadow from dentine, with or without enamel breakdown) or ICDAS 5 (distinct cavity with visible dentine), and who met all eligibility criteria, were enrolled in the study after a detailed explanation about the study by the principal investigator and informed consent and assent were obtained from the parents/primary caregiver and participants. An independent examiner enrolled the participants and randomized them into the treatment methods with teeth assigned to either the CMCR group or the conventional drilling group through simple random sampling.

For both caries removal techniques, theoretical and hands-on training sessions were conducted to ensure consistent and complete caries removal procedures. A pilot study was carried out with five participants (10 teeth) to validate both techniques. These procedures were performed by the principal investigator and verified by a paediatric dentist. The principal investigator and an assistant (serving as an independent examiner) were also trained to assess patient behaviour using the Frankl Behaviour Rating Scale. An inter-examiner reliability score of 0.9 was achieved.

### Sample collection

Prior to treatment, participants performed a pre-procedural oral rinse with normal saline to reduce intraoral debris. Each tooth was isolated using a rubber dam. For the CMCR group, topical anaesthesia was applied to the gingiva, while the conventional group received local anaesthesia. Initial access to the tooth was achieved using a sterile tungsten carbide bur, where necessary. Prior to caries removal, a pre-treatment sample of infected dentine was collected from each tooth using a sterile swab stick, placed in a labelled sterile bottle containing 1.5 ml of normal saline, and transported to the laboratory for microbial analysis. Following caries removal by either method, a post-treatment sample was collected from the prepared cavity for further microbial assessment. Each sample was clearly labelled with assigned identification number per participant, method of caries removal, sampling time (pre or post-treatment). All specimens were delivered to the Microbiology laboratory within one hour for processing.

### Caries removal procedure

The process of caries removal using Papacarie duo gel involved applying the gel to the cavity, waiting for 30 seconds, and removing the softened carious tissue with a blunt spoon excavator. The gel was reapplied until there was no colour change, indicating the cavity was free of infected tissue. Caries removal was assessed and confirmed by the principal investigator utilizing visual and tactile methods. These included observing for brownish discoloration, glazed dentine surface, absence of softness, and non-adherence of a dental explorer to the dentine walls [11]. After confirmation of complete caries removal, a second sample was collected before restoring the teeth with either GIC for primary molars or composite resin for permanent molars. All participants were given postoperative instructions. In the conventional drilling method, a slow-speed handpiece with a tungsten-carbide bur was used to remove carious tissue, under copious irrigation. The drilling was performed intermittently until all softened dentine was removed, while stained but hard dentine was left intact [10]. Visual and tactile methods were similarly used to verify the completeness of caries removal. As with the Papacarie duo gel method, a second sample was collected before restoring the teeth, followed by postoperative instructions. A digital stopwatch was used to record the time (in seconds) taken for each method from the start of caries removal to its completion and confirmation.

### Behavioural assessment

Assessment of participant behaviour using the Frankl behaviour rating scale [12] began from the moment local anaesthesia (for the conventional method) or topical anaesthesia (for the CMCR method) was administered and continued until the caries removal procedure was confirmed as complete. Participants were assigned to a behaviour group only when both the principal investigator and the assistant reached a unanimous agreement. All participants were reviewed one week post-treatment, and none reported any adverse signs or symptoms.

The Frankl scale has four (4) categories ranging from definitely negative to definitely positive. Frankl 1 is a definitely negative category (--) where the child refuses treatment, cries forcefully, is fearful, or exhibits other signs of extreme negativism. Frankl 2, which is a negative category (-) is one in which the child is reluctant to accept treatment, has an uncooperative behaviour, or a mild negative attitude. A child whose behaviour is rated Frankl 3 (+) exhibits a positive, cooperative behaviour, cautiously but willingly accepting treatment, with mild reservations while a child with is rated Frankl 4 (++) being definitely positive). A definitively positively rated child has a good rapport with the dentist, is very interested in the dental procedure, laughs, and enjoys the treatment.

### Sample processing

Bacterial enumeration was carried out using the Total Viable Plate Count method [13]. Bacterial samples were serially diluted across four test tubes labelled 10 ⁻ ¹ to 10 ⁻ ⁴, with each dilution prepared by transferring 1 ml from the previous tube. Subsequently, 1 ml from each dilution was spread on nutrient agar plates, which were incubated for 24 hours to allow bacterial colony formation. Only plates with fewer than 300 colonies were used for calculating the concentration of bacteria in the original sample. The colony-forming units (CFU) were determined by multiplying the number of colonies by the corresponding dilution factor [13]. To eliminate bias, both the principal investigator and the laboratory technician were blinded to whether the samples came from the chemo-mechanical or conventional group.

### Data analysis

Data were processed using Microsoft Excel and analyzed with STATA statistical software. Socio-demographic and clinical characteristics were summarized using frequencies and percentages. The Shapiro–Wilk test was used to assess data normality. The paired t-test was applied to compare pre- and post-treatment bacterial counts within and between groups, as well as time taken for caries removal across the two methods. Frankl behaviour ratings were reported using frequencies, percentages, and medians, and the Wilcoxon signed-rank test was used for comparisons between groups. A 95% confidence interval and *p*-value < 0.05 were considered statistically significant.

### Ethical considerations

Ethics approval for the study (S2 Study protocol in S2 File) was obtained from the AKTH Research Ethics Committee (Reference: AKTH/MAC/SUB/12A/P-3/VI/3229). The primary caregivers of all participants were given a detailed explanation of the study, after which written informed consent to participate and publish the findings of the research was obtained. Child participants also provided assent to participate. The study adhered to the CONSORT reporting guidelines (S1 Consort checklist in S1 File) [14] and was conducted in accordance with the Declaration of Helsinki, following the study protocol. The authors confirm that all related trials for this intervention were registered. After ethics approval and prior to recruiting participants, the clinical trial application for the registration was submitted. Participant enrolment however commenced before the registration approval came due to challenges and unavoidable delays in the registry’s clearance processes. Furthermore, the research was conducted as part of a fellowship program with strict timelines to meet program requirements.

## Results

**Fig 1:**
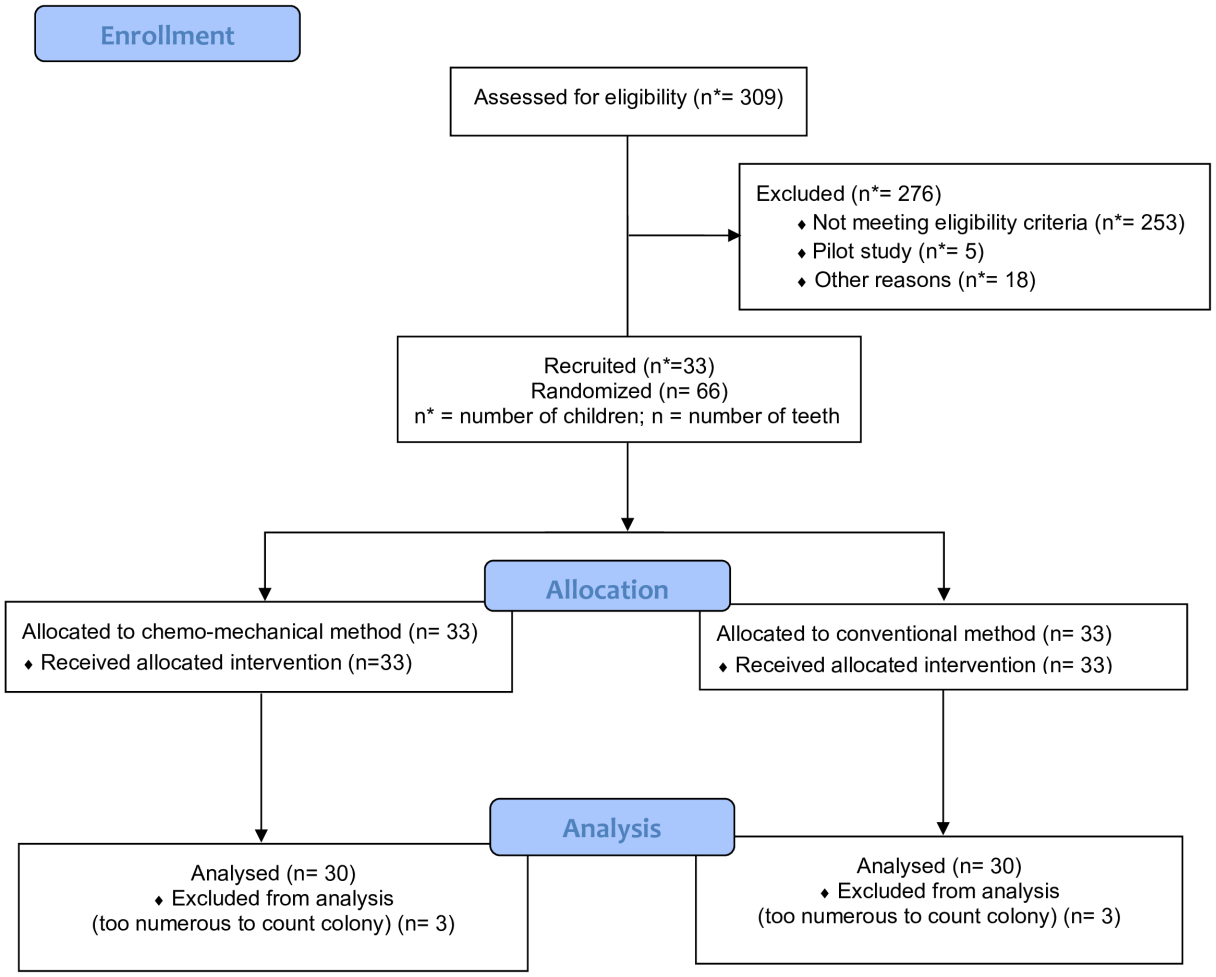
Consort flow diagram of study participants.

Fig 1 shows the Consort flow chart of study participants for their enrolment in the study and the allocation as well as analysis of the teeth and samples, respectively. Of 309 children screened using the eligibility criteria, 276 children were excluded from the study for one reason or the other including being a part of the pilot study, declining consent or not meeting the eligibility criteria.. A total of 33 children (66 teeth) were enroled in the main study. Three samples yielded bacterial colonies that were too numerous to count therefore, the participants associated with those samples were excluded from the final analysis.

Table 1 shows ages of the study participants ranging from 6 to 12 years, with a mean age of 9.2 ± 1.9 years. The mean age for male participants was 9.1 ± 1.9 years, while that of female participants was 9.4 ± 2.2 years. Just over three-fifths of the participants (60.6%) were male, and the remaining 39.4% were female.

**Table 1:** Socio-demographic characteristics of study participants (n = 33).

Age (years)	Female; n = 13 (39.4%)	Male; n = 20 (60.6%)	Total n = 33 (100%)
6	3 (23.1)	1 (5.0)	4 (12.1)
7	0	4 (20.0)	4 (12.1)
8	0	2 (10.0)	2 (6.1)
9	3 (23.1)	4 (20.0)	7 (21.2)
10	3 (23.1)	5 (25.0)	8 (24.2)
11	1 (7.7)	2 (10.0)	3 (9.1)
12	3 (23.1)	2 (10.0)	5 (15.2)
**Mean Age**	9.4 ± 2.2	9.1 ± 1.9	9.2 ± 1.9

Values are frequencies (%).

Table 2 shows the changes in bacterial CFU/ml counts before and after caries removal using both the chemo-mechanical and conventional drilling methods. Intra-group comparisons revealed that the mean bacterial CFU/ml counts were significantly reduced following caries removal with either the chemo-mechanical method (t = 12.47, p < 0.001) or the conventional drilling method (t = 15.43, p < 0.001). However, inter-group comparison showed that teeth treated with the conventional drilling method had a significantly greater percentage reduction in mean bacterial count than those treated with the chemo-mechanical method (t = − 4.42, p < 0.001).

**Table 2:** Comparison of bacteria CFU/ml count within and between treatment groups.

	Chemo-mechanical (n = 30)		Conventional (n = 30)		Intergroup comparison
	Range	Mean (SD)	Range	Mean (SD)	
Before	0.6 – 3.0 × 10^6^	1.6 ± 0.68 × 10^6^	0.56–3.0 × 10^6^	1.7 ± 0.73 × 10^6^	
After	0.0 – 1.6 × 10^6^	0.54 ± 0.40 × 10^6^	0.0–1.5 × 10^6^	0.35 ± 0.36 × 10^6^	t = - 4.42
Reduction	0.46 – 2.4 × 10^6^	1.1 ± 0.47 × 10^6^	0.56–2.1 × 10^6^	1.3 ± 0.47 × 10^6^	*p* < 0.001*
% Reduction	32 – 100	68.6 ± 16.6	49 – 100	82.9 ± 13.9	

Intragroup

Comparison t = 12.47; *p* < 0.001* t = 15.43; *p* < 0.001*.

SD: Standard deviation; t = paired t-test * = Statistically significant.

3 samples from each group were excluded from the analysis due to too numerous to count colony.

Table 3 presents the distribution of time spent on caries removal. The mean time for caries removal using the chemo-mechanical method was 692.4 ± 245.9 seconds (95% CI: 351.6–499.3 seconds), while the mean time for the conventional drilling method was 266.9 ± 101.6 seconds. The difference between the two means was statistically significant (t = 11.73; p < 0.001).

**Table 3:** Time spent (seconds) to complete caries removal between both treatment methods.

Caries removal method	N	Mean (SD)	95% CI	Intergroup comparison
CMCR	33	692.4 ± 245.9	605.1–779.6	*p* < 0.001*
Conventional	33	266.9 ± 101.6	230.9–302.9	t = 11.73
Difference		425.5	351.6–499.3	

SD: Standard deviation; CI: Confidence interval; CMCR: Chemo-mechanical caries removal method.

Table 4 shows that 57.6% and 33.3% of the children treated with the chemo-mechanical method exhibited positive and definitely positive behaviour, respectively, while 66.7% of the children treated with the conventional method demonstrated negative behaviour.

**Table 4:** Association between treatment methods and behaviour based on the Frankl behaviour rating scale.

Treatment Method	Behaviour (%)			Total	χ^2^	*p*-value
	Negative	Positive	Definitely Positive			
Chemo-mechanical	3 (9.1%)	19 (57.6%)	11 (33.3%)	33	**23.45**	< 0.001*
Conventional	22 (66.7%)	6 (18.2%)	5 (15.2%)	33		

Values are frequencies (percentage), n = 33, **χ**^*2*^ = Chi square, * = Statistically significant.

Table 5 shows that during chemo-mechanical treatment, there was no statistically significant association between behaviour and sex (p = 0.125) or age (p = 0.994). Similarly, no significant association was observed between behaviour and either sex (p = 0.924) or age (p = 0.162) during conventional treatment.

**Table 5:** Frankl behaviour rating of children during both treatment methods, by age and sex.

Method	Factor	Total	Behaviour			LRχ^2^	*p*-value
			Negative	Positive	Definitely Positive		
**Chemo-mechanical**							
	**Sex**						
	Female	13	1 (7.7)	7 (53.8)	5 (38.5)	0.266	0.125
	Male	20	2 (10.0)	12 (60.0)	6 (30.0)		
	**Age**						
	<10 years	17	3 (17.6)	12 (70.6)	2 (11.8)	10.278	0.994
	≥10 years	16	0 (0.0)	7 (43.8)	9 (56.3)		
**Conventional**							
	**Sex**						
	Female	13	6 (46.2)	3 (23.1)	4 (30.8)	5.148	0.924
	Male	20	16 (80.0)	3 (15.0)	1 (5.0)		
	**Age**						
	<10 years	17	12 (70.6)	3 (17.6)	2 (11.8)	0.353	0.162
	≥10 years	16	10 (62.5)	3 (18.8)	3 (18.8)		

Values are frequencies (%), n = 33, LR ***=*** Likelihood ratio, χ^2^ ***=*** Chi square.

## Discussion

Treatment of dental caries has always been a challenge, particularly in anxious or apprehensive children [6]. The use of Papacarie as a chemo-mechanical method of caries removal not only improves cooperation among children undergoing dental treatment but also aligns with the principles of minimally invasive dentistry [5]. It is widely accepted that a small amount of residual bacteria left after caries removal is generally harmless and likely to die off once a tight coronal seal is established, cutting off nutrient supply [15]. Most literature agree that complete removal of bacteria prior to restoration is unnecessary and often unachievable [16].

In the current study, a generally high bacterial colony count was observed, which is consistent with the findings of Ammari et al., [17] but contrasts with those of Chowdhry et al., [18] recording bacterial colony counts of 10^5^ and 10^2^, respectively. The elevated bacterial colony counts in this study could be attributed to the high bacterial affinity of the swab sticks used for collecting dentinal/microbial samples [19]. In contrast, many studies [17,20] used spoon excavators or dental burs for sampling, which are less likely to collect sufficient bacteria, particularly after the caries removal process.

The mean total viable count in the current study before caries removal were nearly identical in both treatment groups. A significant reduction in total viable count was observed following caries removal in both the chemo-mechanical and conventional groups, aligning with findings from Singh et al [21]. However, intergroup comparison revealed a significantly higher percentage reduction in the conventional group compared to the chemo-mechanical group. This finding is supported by studies from Aswathi et al. [22] and Almaz et al., [23] who also reported greater bacterial reduction with conventional drilling. Conversely, Ismail et al. [10] reported higher effectiveness of the chemo-mechanical method compared to the conventional approach. A possible explanation for this discrepancy is that Ismail’s study used a ceramic bur (“smart bur”) in the conventional group, which is designed to selectively remove only decayed dentine and not affected or sound tissue. In contrast, the higher reduction in total viable count seen in the conventional group in our study may be attributed to the dental drill’s potential to remove not only infected but also affected and even healthy dentine.

Regarding treatment time, the duration of chemo-mechanical caries removal in this study aligns with previous findings by Balciuniene et al., [24] while the time required for conventional treatment is consistent with that reported by Anegundi et al. [25]. Overall, the chemo-mechanical method took significantly longer, approximately two and a half times longer than the conventional method highlighting its lower efficiency in time use. This finding is in agreement with studies by Bohari et al. [26] and Chowdhry et al. [18] However, Motta et al. [27] and Kotb et al. [28] found no significant time difference between the two methods. The longer treatment time observed in the current study for the chemo-mechanical method may be due to the need for multiple gel applications and the manual excavation involved. Furthermore, a 30-second wait was required after each application to allow for enzymatic degradation of the infected dentine’s partially degraded collagen. Variation in treatment time across studies may also result from differences in the gel application time, which ranges from 20 to 60 seconds depending on the CMCR agent used. Some manufacturers or clinicians may opt for shorter or longer exposure times, contributing to inconsistencies in time efficiency.

The median behaviour rating was “positive” for the chemo-mechanical treatment method and “negative” for the conventional drilling method. This suggests that children generally exhibited willingness and cooperative behaviour during treatment with the chemo-mechanical method, whereas negative behaviour was more frequently observed during the conventional treatment. Interestingly, the children tolerated the longer duration of the chemo-mechanical procedure; without displaying negative behaviour. The good behavior displayed by the children may stem from the fact that no injections were involved in the treatment process. In contrast, the negative behaviours associated with the conventional method were mostly noted during the administration of local anaesthesia (LA) and at the onset of the treatment session. Furthermore, the sound, vibration, and heat generated by dental drills likely heightened fear and anxiety, translating into uncooperative behaviour in some of the children. By comparison, the chemo-mechanical caries removal (CMCR) method does not require LA and does not produce noise or vibration, contributing to more positive patient behaviour during treatment. This observation is consistent with previous research that reported improved behaviour and increased comfort among children treated with CMCR [29,30], and more negative behaviour in those treated with conventional methods [31]. Conversely, Anegundi et al. [25] reported no statistically significant difference in behaviour between the two treatment methods, although children treated with the conventional method experienced more pain. Despite this, most participants in that study still displayed positive behaviour. Our study recorded no statistically significant association between gender or age and children’s behaviour during treatment. This indicates that these demographic factors did not influence behavioural responses. Consequently, the null hypothesis of this study was rejected.

One limitation of this study is that it did not aim to isolate specific microbial species. Instead, all microorganisms present in the carious lesions were cultured collectively. Additionally, although a split-mouth study design was employed, the extent and depth of dentinal caries could not be standardized within the same individual. This variation likely influenced the number of gel applications required per tooth, ultimately affecting the total treatment time. Lastly the study did not separate findings for primary teeth from those of permanent teeth although the research aimed to find alternative treatment for dental caries in children of all ages (having primary and/or permanent teeth).

## Conclusion

This study demonstrated that both the chemo-mechanical and conventional methods of caries removal are effective in reducing bacterial load in carious teeth. While conventional drilling remains the better-established and time-efficient method of caries removal, the chemo-mechanical technique serves as a valuable alternative for managing children, reducing anxiety and promoting greater comfort and cooperation during treatment.

## Recommendation

While the study supports the superior efficacy and time efficiency of the conventional drilling method, it recommends the use of chemo-mechanical agents as an alternative, especially when managing children with uncooperative or anxious behaviour. Moreover, the philosophy of minimally invasive dentistry aligns with the use of CMCR, as it effectively reduces microbial load while preserving tooth structure.

## Supporting information

S1 File CONSORT checklist. (DOCX)

S2 File Study Protocol. (DOCX)

S3 File Minimal Dataset. (XLSX)
