# Supplementary material for: Comparison of chemo-mechanical and conventional caries removal methods in 6-to-12-year-old children: Randomized clinical trial
Source: PLoS One. 2026 Jan 16;21(1):e0339771. doi: 10.1371/journal.pone.0339771 (PMC12810845; doi:10.1371/journal.pone.0339771)
Supplement: S2 File — (DOCX) [file pone.0339771.s002.docx]

A split – mouth Randomized Clinical Trial to investigate the effect of two caries removal methods in a paediatric population in Nigeria: Protocol and study design

MALAMI A.B.^1^, ADEYEMO Y.I.^1^, AMINU A.^2^, OREDUGBA F.A.^3^

^1^Department of Child Dental Health, Bayero University, Kano. Kano State, Nigeria. ^2^Department of Medical Microbiology, Bayero University, Kano. Kano State, Nigeria.

^3^Department of Child Dental Health, Lagos University Teaching Hospital, Lagos State, Nigeria.

Corresponding author:

Yewande Isabella Adeyemo

Department of Child Dental Health,

Bayero University, Kano. Kano State, Nigeria.

[wendiepee@gmail.com](mailto:wendiepee@gmail.com)

Abstract

**Background**: Dental caries is a public health burden in most countries across the world, and it is the leading cause of tooth loss among the paediatric age group. The most popular method of caries removal in a tooth prior to restoration is by drilling, using handpieces and burs (conventional method). In this method, all carious teeth are treated by elimination of both infected and affected dentine. Another method of caries removal is by the use of chemo-mechanical agents. These chemical agents not only selectively identify and remove infected dentine tissues but give a chance for remineralization of affected dentine hence, preserving healthy tissues. Hence, the need to evaluate and compare these two approaches, particularly in light of the evolving trend towards minimal intervention dentistry.

**Aim**: To evaluate and compare the effectiveness of caries removal, using the chemo-mechanical agent (papacarie duo gel) and the conventional rotary instrument in primary and permanent molar teeth of children aged 6 to 12 years in the Paediatric Dentistry Clinic of Aminu Kano Teaching Hospital Kano, Kano State.

**Methods/design**: The study is designed as a split mouth randomized clinical trial, consisting of 66 molar teeth selected from 33 healthy children. Papacarie duo gel will be used as the chemo-mechanical agent and enumeration of bacteria will be done by “Total viable plate count”. Effectiveness of caries removal using caries detector dye (CDD), participants’ behaviors and treatment preferences, as well as time taken to complete caries removal will be assessed and compared. The participants will have a longitudinal clinical follow up of the restorations for two years, where retention and marginal leakage in the two treatment groups will be assessed.

**Discussion**: If this treatment approach proves effective, it has the potential to reduce the number of untreated dental caries, especially in paediatric age group in similar settings.

**Trial registration**: PanAfrican Clinical Trial Registry ID: PACTR202310877678533. Registered on 10^th^ October 2023.

**Keywords**: Dental caries, Chemo-mechanical caries removal, Conventional drilling, Paediatric dentistry, Aminu Kano Teaching Hospital.

## Introduction

Dental caries is a common oral disease with significant medical, social, and economic consequences, particularly affecting low-income countries and disadvantaged populations.^1^ Factors contributing to the formation of dental caries include socio-economic status, gender, age, dietary habits, oral hygiene, and inadequate utilization of health services.^2^ This condition is a primary reason for parents to seek professional care for their children in paediatric dental clinics.^3^ Often patients present with lesions requiring operative intervention, such as cavitated caries or failed restorations.^4^

The prevalence of dental caries has decreased notably in many nations, particularly in high-income countries like the United States and Europe, with a reported reduction of 90% in decayed, missing, and filled teeth (dmft) in 5-12 year-olds.^5^ However, this decline is less pronounced in low-income countries.^6^ A U.S. study by Dye et al. revealed that approximately 37% of children aged 2-8 have experienced dental caries in their primary teeth, with 14% having untreated caries.^7^ In permanent teeth, 21% of children aged 6-11 had caries experience, with around 6% having untreated caries.^7^ In Africa, there has been a shift in caries prevalence, attributed to increased sugar consumption and inadequate fluoride exposure.^8^ In Nigeria, dental caries prevalence ranges from 4-30%, with rates of 17.9% in Lagos, 15.7% in Benin, and 24.1% in Enugu among children.^9,10^ Ojukwu *et al*. reported a prevalence of caries in Kano as 22.4% with a mean dmft/DMFT score of 0.21±0.41/0.14±0.35.^11^ The authors also reported that, among the 4-to-17-year-olds, the prevalence was higher in primary than permanent dentition, and higher in females than males.^11^ Studies also indicate higher prevalence in females and differences between urban and rural areas, as well as between private and public schools.^11^ Overall, dental caries prevalence varies within countries and among different demographic and socioeconomic groups.^12^

Paediatric dentists are faced by numerous challenges when managing dental caries, particularly when the decay extends into dentine, necessitating removal of decayed dentine before tooth restoration.^13^ Traditional methods involve mechanical drilling, which has drawbacks such as patient fear, anxiety, and the risk of disease transmission through aerosols generated by the drill, including COVID-19.^14,15^ The Caries Management by Chemo-mechanical Removal (CMCR) technique offers an alternative by using chemical agents to dissolve decayed tissue, eliminating the need for anesthesia and reducing the risk of aerosol transmission.^16^ The shortcomings of this technique of caries removal however include the use of dental drills which may still be needed to remove overhanging enamel in order to gain access to some cavities.^16^ While CMCR shows promise, its efficacy needs verification, especially in Nigeria, where data is lacking.

In summary, CMCR has the potential for improvement in the treatment approach for dental caries, especially in the paediatric age group that will be achieved with reduction in the number of untreated dental caries.^17^ This method of caries removal stands a chance of creating a patient and operator friendly environment that is less associated with anxiety, fear and transmission of diseases.

This research study aims to evaluate and compare the effectiveness of caries removal, using the papacarie duo gel (chemo-mechanical method) and the conventional rotary instrument in primary and permanent molar teeth of children aged 6 to 12 years in the Paediatric Dentistry Clinic of Aminu Kano Teaching Hospital Kano, Kano State.

## Specific objectives

- - - 1. To assess and compare the effectiveness of papacarie duo gel and conventional drilling methods of carious tissue removal using bacterial colony forming unit (CFU) count in both primary and permanent molar teeth, as well as using caries detector dye.
      2. To determine and compare the time taken to complete caries removal when using the papacarie duo gel and conventional drilling methods.
      3. To assess and compare the participants’ preference for the treatment methods as well as their behaviour during treatments using Frankl behaviour rating scale.
      4. To evaluate the clinical retention and marginal leakage of restorations in teeth treated with papacarie duo gel method and conventional drilling method.

Research Hypothesis

Null:

The effectiveness of caries removal using papacarie duo gel (chemo-mechanical method) and conventional drilling methods of caries removal in molar teeth will not differ.

Alternate:

The effectiveness of caries removal using papacarie duo gel (chemo-mechanical method) and conventional drilling methods of caries removal in molar teeth will differ, and the difference will be statistically significant.

**Methods/design**

## Study Location.

The study location will be the Paediatric Dentistry Clinic of the Department of Child Dental Health, Aminu Kano Teaching Hospital (AKTH) Kano, Kano State, Nigeria. Kano is the third largest city in Nigeria after Lagos and Ibadan, covering a total area of 20,131 km^2^, with a GDP of US$12,393 as at 2010. It is the most populous state in the country with a total population of 9,401,288 and metro area population of 2,958,000 based on the 2006 National Population Census.^18^ The United Nations population projection for Kano city in the year 2021 was 4,103,000.^19^ AKTH is a tertiary hospital that was established in August 1988 and has a bed capacity of about 700. The hospital serves as a major referral centre for the neighbouring states of Katsina, Jigawa, Bauchi and Zamfara. The Paediatric dentistry unit of Aminu Kano teaching hospital frequently attends to patients with dental caries and its complications. The unit has two consultants, three senior registrars, four registrars and three dental surgery technicians.

## Study Design.

The study design is a split mouth randomized clinical trial, where two treatment options of caries removal will be carried out in each participant. The effectiveness of chemo-mechanical (papacarie duo gel) and conventional drilling methods of caries removal will be evaluated and compared in participants aged 6 to 12 years before restoring the affected teeth using Glass Ionomer Cement (GIC) in primary teeth and composite resin in permanent teeth. A 2-year longitudinal clinical follow up of the participants will be done so their restorations can be assessed. The SPIRIT reporting guideline^20^ was used in the development of the trial protocol.

## Study Population.

The study population will consist of 6-to-12-year-old children presenting to the Paediatric Dentistry clinic of AKTH, Kano.

## Inclusion Criteria

Children between the ages of 6 and 12 years (Mixed dentition stage).

Children who have at least two carious teeth with ICDAS score 4 or 5 on primary or permanent molars.

Children with Class I carious lesions (occlusal caries).

Children who give their assent and whose parents give consent to participate in the study.

## Exclusion Criteria

- - - 1. Children who have teeth with clinical signs and symptoms of pulpitis, periodontitis, pulpal necrosis, fistula, or abscess.
      2. Children with medical conditions, chronic illnesses or on long term medication.
      3. Children with ICDAS score of 4 or 5 for a molar tooth that is mobile, unrestorable, or has arrested caries.
      4. Children who have teeth with developmental anomalies such as molar incisor hypomineralization, dental fluorosis, taurodontism, germination, fusion etc.
      5. Children with recurrent caries or failed restoration.
      6. Highly uncooperative children.
      7. Children whose primary molars are close to exfoliation as identified from the radiograph.

## Study Duration.

The study will commence in February 2022 and will be completed after obtaining the required sample size and following up with participants for two years after treatment.

## Sample Size Determination.

The estimated sample size of participants for this split – mouth randomized control trial will be determined using the sample size formula for comparative studies^21^ and the means and standard deviations from a previous study.^22^

Calculation:

n = [(Z_α/2_ + Z_β_)^2^ x {2(σ1 − σ2)^2^}]

(µ_1_ - µ_2_)^2^

Where

n = minimum sample size required in each group.

Z_α/2_ = Probability that if the two groups differ, this reflects a true difference in the two populations otherwise known as the confidence level. This has been set at 95% level corresponding to 1.96 from the normal distribution table.

Z_β_ = This is the power of the study. It is the probability that if the two populations differ, the samples would show a significant difference. For the study it is set at 80% equivalent to 0.842 on the normal distribution table.

Using the reference article on evaluation of the efficacy of caries removal,^22^

ơ1 = Standard deviation of the chemo-mechanical (papain-based gel) method of caries removal group (Group 1). That is = 87,000.

ơ2 = Standard deviation of the conventional method of caries removal group (Group 2). Which is = 98,000.

µ_1_ = mean deviation of Group 1. That is =111,000.

µ_2_ = mean deviation of Group 2. That is =119,000.

Substituting the values obtained from a previous similar study,

n *=* (1.96 + 0.842)^2^ x 2(87,000 – 98,000)²

(111,000 **–** 119,000)^2^

n = 29.7, that is approximately 30 teeth for each treatment group (minimum).

Accounting for a 10% non – response rate (N = N/1 – r), this yields a minimum sample size of 33 teeth for each treatment group, giving a total of 66 teeth in the study. This translates to 33 children, with each child each having two comparable carious teeth on either side of the mouth (split – mouth).

**Consent Procedure and Ethical Considerations**

The primary caregivers of selected participants will be given a detailed explanation of the study and written consent will be obtained for their children/wards to participate in the study while assent will be obtained from the participants before recruitment into the study. Data to be collected will be treated with utmost confidentiality. Codes will be substituted for patients’ names as a means of identification. All collected data will be transferred to a password protected personal computer and all published articles arising from this research will bear no information that reveals the identity of the participants. Participants who meet the inclusion criteria and require other medical or dental care will be treated accordingly or referred to the appropriate specialists at the Aminu Kano Teaching Hospital detailing their exact needs. Those who meet the inclusion criteria but decided not to partake in the study will be treated without prejudice. Participants will only pay half the sum of the cost for all the services rendered to them, while the remaining half including microbiology laboratory charges will be incurred by the principal investigator. Participants retained the right to decline participation in the study, or to withdraw at any time during the course of the study without any loss of benefit or reduction in the quality of care receivable at the Paediatric Dentistry clinic or at the Aminu Kano Teaching Hospital Community Dentistry Outpost-clinic. Any subject that declines participation will not be included in the study. This study will be conducted in accordance with the Declaration of Helsinki and adhere strictly to the WHO treatment and infection control protocols that ensure the use of only sterile instruments and the protection of all patients from cross-infection.

## Sampling Technique.

The teeth will be randomly allocated to one of the two treatment methods (either the CMCR method or the conventional drilling method) by asking the participants to choose heads or tails on a coin. Thereafter, a coin will be tossed. If it lands on heads, the tooth on the right side receives the CMCR method, and the tooth on the left side receives the conventional method. If it lands on tails, the tooth on the left side receives the CMCR method, and the tooth on the right side receives the conventional treatment method.

## Description of Instrument.

## Data Collection Form.

The study instrument for the data collection consists of five sections:

1. For Biodata and socio-demographic information.
2. For carious tissue samples (before caries removal and after caries removal).
3. For evaluation of effectiveness (complete or partial) of caries removal in both treatment groups using caries detector dye.
4. For time taken to complete the caries removal process.
5. For assessment of the participants’ preference for the treatment methods.
6. For assessment of the participants’ behavior during both treatment methods using Frankl behaviour rating scale.
7. For evaluation of retention and marginal leakage of restorations in participants treated with chemo-mechanical agent and conventional rotary instrument using codes and criteria used in assessing survival of one-surface restoration by Phantumvanit et al.^23^

## Armamentarium.

Instruments will be sterilized by autoclaving and single-use materials will be discarded after use.

Instruments, equipment, and materials used include:

1. Pana max Turbine handpiece (NSK, Tochigi-ken, Japan).
2. Slow speed (contra-angle) handpiece (NSK, Georgia, USA).
3. Examination set (dental mirror and round ended explorer).
4. College tweezer.
5. No 3 round carbide bur and inverted cone bur.
6. 1.5mm stainless steel spoon excavator.
7. Papacarie duo gel.
8. Rubber dam kit (Coltene Whaledent, Altstatten, Switzerland).
9. Lidocaine HCl, epinephrine 1:80000 (Laboratorios Zeyco, Zapopan, Mexico).
10. Dental syringe.
11. 10% xylocaine pump spray/lidocaine 10 mg/dose (Astrazeneca AB 15185 Sodertalje, Sweden).
12. Sterile swab stick (sterile polyester tipped applicator).
13. Caries-detector dye.
14. Radiopaque Posterior Glass Ionomer Restorative Cement, GC Gold Label, High Strength posterior restorative material. (GC Corporation Tokyo, Japan).
15. Petroleum jelly (Vaseline).
16. Unisal 0.9% normal saline (Unique Pharmaceuticals Ltd, Sango Ota, Nigeria).
17. RubyComp Nano Hybrid Composite (Nano Hybrid Dental Material Kit, Turkey).
18. Sterile bottle.
19. Ice box.
20. Pipette.
21. Nutrient agar.
22. Glass tube.
23. Biohazard bag.
24. Vortexer machine.
25. Film for periapical radiograph.

## Recruitment.

Participants for this study will be recruited from the Paediatric Dentistry clinic of the Aminu Kano Teaching Hospital, Kano. A detailed history will be obtained from each participant and from the accompanying adult. The principal investigator and an assistant will conduct an oral examination and assessment of each participant on a dental chair with the light on, using a sterile dental mirror and explorer. Pre-treatment radiographs will be taken and documented. Patients having caries with an ICDAS score of 4 (underlying dark shadow from dentine with or without enamel breakdown) or 5 (distinct cavity with visible dentine) and have fulfilled the eligibility criteria will be recruited into the study after full explanation and assent and informed consent obtained from the patient and parent/caregiver, respectively (APPENDIX A).

## Calibration and Inter-Examiner Reliability Test.

For the clinical intervention (caries removal in both treatment methods), theoretical and practical sessions will be conducted, where complete caries removal is to be discussed exhaustively. A pilot study will then be carried out on five participants (10 teeth), where complete caries removal will be performed by the principal investigator using both the CMCR and conventional methods, which will be validated by a Paediatric dentist.

Two independent examiners will be trained on using codes and criteria by Phantumvanit for the assessment of retention and marginal leakage of restorations.^23^ The principal investigator and an assistant will also be trained to assess patient behaviour using the Frankl Behaviour Rating Scale. An inter-examiner reliability score will then be calculated.

## Sample Collection

Pre-procedural oral rinse using normal saline will be performed by all participants in order to reduce debris in the oral cavity. Each tooth in both groups will be isolated with the aid of a rubber dam, after application of topical anaesthesia to the gingivae to reduce discomfort associated with placement of rubber dam clamp in CMCR group, and after administration of local anaesthesia in relation to each tooth for cavity preparation using drilling method. Initial access will be made, in instances where the teeth have overhanging enamel, with the aid of a tungsten carbide round bur on a high-speed handpiece.

Before commencement of caries removal using both methods, sterile swab sticks will be introduced into the cavities to take samples of decayed/softened dentine tissue from the walls and floor of each carious tooth. Each sample collected with a swab stick will be placed into an appropriately labelled sterile bottle containing 1.5mls of normal saline and transferred into an icebox for transportation to the laboratory. Thereafter, removal of carious tooth tissue will be carried out via the CMCR and the conventional drilling methods independently. Both treatment methods will sequentially be followed immediately after the first sample was collected for microbial analysis. After this, a second sample will immediately be collected from the cavity walls and floor in both treatment methods using a sterile swab stick for assessment of microorganisms that may have escaped the caries removal process. Each swab stick will also be placed into a labelled sterile bottle containing 1.5mls of normal saline, which will be transferred into the icebox alongside previous samples taken, for preservation. All samples collected will be labelled using participants’ identification, indicating either before or after caries removal and the method of caries removal used. The samples will be taken to the microbiology laboratory within one hour of collection for microbiological assessment.

## CMCR (Papacarie Duo) Method.

After collecting the first sample of carious/softened dentine, caries will be removed using the papacarie duo gel according to the manufacturer’s instructions. The gel will be syringed directly into the cavity, until all carious lesions are completely covered by the gel and left for a duration of 30 seconds after application. After 30 seconds, the gel will become turbid and contaminated with debris, indicating that the caries removal process can be started. Softened carious tooth tissue will be removed using a sterile blunt spoon excavator in a pendulum motion without applying excessive pressure on the cavity walls and floor. The gel will be applied repeatedly until no significant change in colour is observed of the gel, suggesting the cavity is free of infected tissue. Complete caries removal will be assessed by the principal investigator and confirmed/validated by a paediatric dentist utilizing visual and tactile methods. These include brownish discoloration of the dentine, glazy appearance of the dentine, absence of softened dentine and the dental explorer not sticking to the dentine walls.^24^ When complete caries removal is confirmed, a second sample will then be collected. All procedures will be performed under the principles of good clinical practice.

## Conventional Drilling Method.

After the collection of the first carious/softened dentine sample, all carious tissue will be removed using a sterile inverted carbide bur on a slow speed handpiece. Tungsten-carbide bur will be used as this helps to reduce the risk of excessive tissue removal.^21^ The drilling process will be done intermittently until all softened dentine is removed, while stained hard dentine will be left untouched. Visual and tactile methods of assessment for complete caries removal will be employed as in the CMCR method. When complete caries removal is confirmed, a second sample will then be collected.

## Evaluation of Effectiveness of Caries Removal using Caries-detector Dye.

After caries removal is confirmed and a second sample collected, the cavity will be cleaned and dried using compressed air. Caries-detector dye will be applied to each of the cavity walls and floor for a period of 10 seconds. The cavity will thereafter be rinsed thoroughly with water and dried using compressed air. Any tooth with dentine that deeply retained dye will be marked as incomplete caries removal, while dentine that lightly/sparsely retained dye or did not retain dye completely, will be marked as a cavity that have complete removal of caries.^25^ After the caries detector dye process, each cavity will be cleaned with sterile moistened cotton pellets, dried, and restored with GIC or composite resin for a primary molar or permanent molar tooth, respectively.

## Evaluation of Cavity Preparation Time.

A digital stopwatch will be used to assess the time taken from the beginning of the caries removal process to when it will be completed and confirmed, measured in seconds for each method. An assistant who is dedicated to the task will do this assessment.

**Participants’ Preference for the Treatment Methods as well as their Behaviour During Treatments using Frankl Behavior Rating Scale.**

At the end of the treatments, all participants will be interviewed on their preferred method of treatment, and their responses will be recorded. The participants’ behaviours will be assessed by both the principal investigator and the assistant using the Frankl Behaviour Rating Scale, starting from the time local or topical anaesthesia is administered until the tooth is restored.

Frankl behavior rating scale assesses participants’ behaviours as follows:

**Frankl 1 (definitely negative)**: Refusing treatment, crying forcefully, fearfulness, any other evidence of extreme negativism.

**Frankl 2 (negative)**: Reluctant to accept treatment, uncooperative behavior, some evidence of negative attitude but not pronounced.

**Frankl 3 (positive)**: Acceptance of treatment, at times cautious, willingness to comply with dentist, reservations but follows dentist directions cooperatively.

**Frankl 4 (definitely positive)**: Good rapport with dentist, interested in dental procedure, laughing and enjoying procedure.

## Evaluation of Retention and Marginal Leakage of Restorations (success of restorations) in Teeth Treated with Chemo-mechanical Agent (papacarie duo) and Conventional Rotary Instrument.

One month after the treatment is completed, participants will be recalled, and all restorations will be assessed according to codes and criteria used by Phantumvanit *et al*.^23^ for survival rates of the restorations. The follow up examinations will be performed by an independent examiner who is blinded to the caries removal procedure the participants received. The examination will be carried out on a dental chair with the light on using a 0.5mm ball ended CPITN (WHO) probe and a mouth mirror. According to the criteria used, codes 0 and 1 are considered acceptable (successful) restoration and codes 2, 3, 4, 5 and 6 are considered unacceptable (failed) restorations, while code 9 is excluded since restoration cannot be diagnosed. (APPENDIX B). A repeat assessment using the same criteria will be carried out at 3 months, 6 months, 1 year and 2 years after the initial restoration.

## Sample Processing.

Enumeration of bacteria will be done by “Total viable plate count”.^26^ To disperse bacterial aggregates, each sterile bottle containing samples of carious tissue in a swab stick will be agitated in a vortex machine to ensure an even distribution of bacteria. Four tubes containing 9ml saline will be labelled 10^-1^, 10^-2^, 10^-3^, and 10^-4^, respectively. Four nutrient agar plates will also be labelled 10^-1^, 10^-2^, 10^-3^, and 10^-4^, respectively. One ml will aseptically be removed from the original sample with a sterile pipette and transferred to the 10^-1^ dilution tube to make 1:10 dilution. After the 10^-1^ tube is vortexed, 1ml will be transferred from it to 10^-2^ tube making a 1:100 dilution. Again the 10^-2^ dilution tube will be vortexed, and 1 ml will be transferred from it to 10^-3^ tube making 1:1000 dilution. The 10^-3^ dilution tube will be vortexed and 1ml will be transferred to 10^-4^ tube and vortexed, making 1:10,000 dilutions.^26^ Using a new sterile pipette, 1ml will aseptically be transferred from the suspensions and poured onto the surface of a nutrient agar plates. The plates will afterward be incubated aerobically for 24 hours, such that colonies formed can be seen without magnification and the number of bacteria in the original sample can be determined by counting the number of colonies.^26^ In the laboratory, the principal investigator will be blinded to whether the samples belonged to the chemo-mechanical or conventional treatment method.

## Viable Plate Counts Steps.

1. After incubation, the colonies on each of the plates will be counted. Holding the plate to a light source, the colonies will be counted by marking their position on the back of the nutrient agar plates with a marking pen. This aids in keeping track of the colonies previously counted and prevents recounts. All plates with more than 300 colonies will be recorded as TNTC (too numerous to count).^26^

2. From the plate count data, the concentration of bacteria in the original sample will be calculated. For statistical reasons, only data from plates that have less than 300 colonies in this calculation will be used. Each colony-forming unit (CFU) represents a single cell, or a group of cells attached together and inseparable by shaking. Therefore, the number of CFU in the original sample will be determined by multiplying the number of colonies by the dilution factor used. For example, if there are 200 colonies on the plate poured using a 10^-4^ dilution factor, there would be 200 x 10,000 = 2,000,000 colonies or 2 x 10^6^ CFU/ml in the original sample.^26^

## Data Analysis

At the end of data collection, data will be processed in Microsoft Excel, while analysis will be performed using STATA statistical software (Release 15, StataCorp 2017). Socio-demographic and clinical characteristics will be summarized using frequencies and percentages. Data normalcy will be tested by the Shapiro-Wilks test and any value greater than 0.05 will be considered normally distributed. The difference between the pre-treatment bacterial count and post-treatment count in each group (within group difference) and intergroup comparison of bacterial counts will be tested using paired t-test. Furthermore, the mean difference in time taken for caries removal in both treatment groups will be tested using paired t-test**.** The difference in effectiveness of caries removal using caries detector dye and mean difference in clinical survival rates of restoration (retention of restorative material & marginal leakage) between the two treatment groups will be tested for significance using McNemar’s Chi-square test**.** A 95% Confidence Interval will be used to confirm if a relationship exists between the variables. Frankl’s behavior ratings will be compared between the two methods using the Wilcoxon signed-rank test. The level of significance is set at p<0.05.

## Dissemination and publication

Participants and caregivers will be informed about the findings of this research study. The research findings will also be published in scientific journals.

**Discussion**

The efficacy of chemo-mechanical gels (papacarie duo) have purportedly been established in different parts of the world, however there is paucity of data in Nigeria that establishes the efficacy of these chemical agents. This prompts the need to conduct a study on the effectiveness of these agents in our environment so that a possible alternative to the conventional drilling method can be considered and also generate data that can be compared to global standards. This study also seeks to bring to the fore, the concept of minimally invasive dentistry and by extension, atraumatic restorative treatment (ART) particularly in our setting where we lack dental services and the services of dental professionals and specialists. If proven effective, CMCR will help dental care providers offer cost-effective services to children with dental fear and anxiety. Therefore, more evidence-based testing is needed to show that these products are clinically reliable. More so, information on the clinical and microbiologic effectiveness of the use of conventional rotary methods of caries removal in Nigeria is lacking. Therefore, this study shall provide the necessary baseline data that will assist relevant authorities to develop a policy at the national level and treatment protocol in the Paediatric dentistry clinic of AKTH as well as in other clinical settings.

**Authors’ contribution**

ABM is the principal investigator and the lead in developing the trial protocol for this research work. YA, AA, and FO contributed to the study design and data analysis sections. All authors reviewed, read and approved the final manuscript.

## Ethics declarations

Ethical approval for this study was obtained from Aminu Kano Teaching Hospital Research Ethics Committee vides AKTH/MAC/SUB/12A/P-3/VI/3229.

## Funding

The principal investigator will solely finance this project.

**Declaration of competing interest**

The author declares no conflict of interest.

**Data availability**

Request for data will be considered on case-by-case basis and according to the data privacy rules of the Nigerian government.

**References.**

- - - 1. Rugg-Gunn A. Dental caries: strategies to control this preventable disease. Acta Med Acad. 2013;42(2):117–130.
      2. Mulu W, Demilie T, Yimer M, Meshesha K, Abera B. Dental caries and associated factors among primary school children in Bahir Dar city: a cross-sectional study. BMC Res Notes. 2014;7(949):1–7.
      3. Olatosi OO, Onyejaka NK, Oyapero A, Ashaolu JF, Abe A. Age and Reasons for First Dental Visit Among Children in Lagos, Nigeria. Niger Postgrad Med J. 2019;26(3):158–163.
      4. Dhamija N, Pundir PD. A Review on Agents for Chemo-mechanical Caries Removal. Sch J Dent Sci. 2016;3(9):264–268.
      5. Kassebaum NJ, Smith AGC, Bernabé E, Fleming TD, Reynolds AE, Vos T, et al. Global, Regional, and National Prevalence, Incidence, and Disability-Adjusted Life Years for Oral Conditions for 195 Countries, 1990-2015: A Systematic Analysis for the Global Burden of Diseases, Injuries, and Risk Factors. J Dent Res. 2017;96(4):380–387.
      6. Frencken JE, Sharma P, Stenhouse L, Green D, Laverty D, Dietrich T. Global epidemiology of dental caries and severe periodontitis – a comprehensive review. J Clin Periodontol. 2017;44(18):94–105.
      7. Dye BA, Thornton-Evans G, Li X, Iafolla TJ. Dental caries and sealant prevalence in children and adolescents in the United States, 2011-2012. NCHS Data Brief. 2015; 191:1–8.
      8. Veiga N, Pereira C, Amaral O. Prevalence and Determinants of Dental Caries in Portuguese Children. Procedia - Soc Behav Sci. 2015; 171:995–1002.
      9. Oziegbe EO, Esan TA. Prevalence and clinical consequences of untreated dental caries using PUFA index in suburban Nigerian school children. Eur Arch Paediatr Dent. 2013;14(4):227–231.
      10. Folayan MO, Chukwumah NM, Onyejaka N, Adeniyi AA, Olatosi OO. Appraisal of the national response to the caries epidemic in children in Nigeria. BMC Oral Health. 2014;14(76):1–10.
      11. Ojukwu BT, Balarabe SA, Akhiwu BI. Assessment of dental caries prevalence, severity and consequences among institutionalized orphans in Kano state, Nigeria. Int J Med Health Res. 2019;5(7):51–55.
      12. Onyejaka NK, Olatosi OO, Ndukwe NA, Amobi EO, Okoye LO, Nwamba NP. Prevalence and associated factors of dental caries among primary school children in South-East Nigeria. Niger J Clin Pract 2021; 24:1300-1306.
      13. Lula ECO, Monteiro-Neto V, Alves CMC, Ribeiro CCC. Microbiological analysis after complete or partial removal of carious dentin in primary teeth: A randomized clinical trial. Caries Res. 2009;43(5):354–358.
      14. Appukuttan DP. Strategies to manage patients with dental anxiety and dental phobia: literature review. Clin Cosmet Investig Dent. 2016; 8:35–50.
      15. Rothan HA, Byrareddy SN. The epidemiology and pathogenesis of coronavirus disease (COVID-19) outbreak. J Autoimmun. 2020; 109:1 – 4.
      16. Ganesh M, Parikh D. Chemomechanical caries removal (CMCR) agents: Review and clinical application in primary teeth. J Dent Oral Hyg. 2011; 3:34–45.
      17. Singhal DK, Acharya S, Thakur AS. Microbiological analysis after complete or partial removal of carious dentin using two different techniques in primary teeth: A randomized clinical trial. Dent Res J (Isfahan). 2016;13 (1):30–37.
      18. Government notice- Federal Republic of Nigeria Official Gazette. Report on the census 2006 final results. 2009. <https://gazettes.africa/archive/ng/2009/ng-government-gazette-dated-2009-02-02-no-2>.
      19. Demographic statistics bulletin 2017. National Bureau of statistics, May 2018. (https://www.macrotrends.net>cities>kano>population).
      20. Chan A-W, Tetzlaff JM, Gøtzsche PC, et al. SPIRIT 2013 explanation and elaboration: guidance for protocols of clinical trials. BMJ 2013; 346: e7586.
      21. Neilson T. King of charcoal: Japanese create new life for dying industry. Inwood Mag. 2011; II (96):32–33.
      22. Ismail MMM, Al Haidar AHMJ. Evaluation of the efficacy of caries removal using papain gel (Brix 3000) and smart preparation bur (in vivo comparative study). J Pharm Sci Res. 2019;11(2):444–449.
      23. Phantumvanit P, Songpaisan Y, Pilot T, Frencken JE. Atraumatic Restorative Treatment (ART): a three-year community field trial in Thailand-survival of one-surface restorations in the permanent dentition. J Pub Health Dent. 1996;56(3):141-145.
      24. Simón-Soro A, Mira A. Solving the etiology of dental caries. Trends Microbiol. 2015;23(2):76–82.
      25. Umeda JE, Chichakly K, Passos GF, Terada RSS, Pascotto RC, Fujimaki M. System dynamics modeling for tooth decay treatment in Brazilian children. Braz Oral Res. 2020;34: 1–8.
      26. Bassiri E, RejaneBorges M. Microbiology BIOL 275. In: Microbiology Biol 275 enumeration of microorganisms. Academia; 1–10.

**APPENDIX A**

**STATEMENT OF PERSON OBTAINING INFORMED CONSENT.**

I have fully explained the research to………………………………………….. and given sufficient information, including the risks and benefits to make an informed decision.

Date......................................... Signature………………………………….

Name…………………………………………………………….

**STATEMENT OF PERSON GIVING CONSENT.**

I have read the descriptions of the research and discussed it with the doctor to my satisfaction. I fully understand that my participation is voluntary and I know enough about the purpose, methods and risks as well as its benefits. I understand that I may freely withdraw my participation at any time I wish. I have a copy of this consent form and additional information to keep for myself.

Date ……………………………….. Signature…………………………………..

Name………………………………………………………………..

**ASSENT OF THE CHILD**

Do you understand this research study and are you willing to take part in it? Yes [ ] No [ ]

Has the researcher answered all your questions? Yes [ ] No [ ]

Do you understand that you can pull out of the study at any time? Yes [ ] No [ ]

_________________________ ____________________

Signature of Child Date

For further enquiries, please contact the Researcher:

Dr. Abdulrahman Bala MALAMI

08069661989

[malamidental@yahoo.com](mailto:malamidental@yahoo.com)

Child Dental Health Department

(Paediatric Dentistry Unit)

Aminu Kano Teaching Hospital, Kano.

**APPENDIX B**

Data collection form for the study.

COMPARATIVE EVALUATION OF CARIES REMOVAL METHODS IN 6 TO 12 YEAR OLD IN KANO USING CHEMOMECHANICAL AGENT OR CONVENTIONAL ROTARY INSTRUMENT.

**Section A - Biodata**

Name:

Age (as at last birthday):

Sex:

Ethnicity:

**Section B: Collection of carious dentine samples.**

Carious tooth:

ICDAS classification:

| Dentine sample in  Conventional method | Before caries removal  (Quantity of bacteria in unprepared cavity) | After caries removal  (quantity of bacteria  in prepared cavity) | Difference in quantity of bacteria. |
| --- | --- | --- | --- |
|  |  |  |  |

| Dentine sample in  CMCR Method | Before caries removal  (Quantity of bacteria in unprepared cavity) | After caries removal  (quantity of bacteria  in prepared cavity) | Difference in quantity of bacteria |
| --- | --- | --- | --- |
|  |  |  |  |

**Section C: Assessment of complete caries removal using caries detector dye.**

1. Complete caries removal
2. Incomplete caries removal

**Section D: Time taken to complete caries removal in seconds.**

**a.** Conventional method **----------------**

b. Chemo-mechanical method **----------------**

**Section E: Assessment of participants’ behaviour during treatment using Frankl behaviour rating Scale.**

**Frankl Behaviour Rating Scale:**

1. **Frankl 1 (Definitely Negative):** Refusal of treatment, forceful crying, fearfulness, or other signs of extreme negativism.
2. **Frankl 2 (Negative):** Reluctance to accept treatment, uncooperative behaviour, or mild negative attitude.
3. **Frankl 3 (Positive):** Cautious acceptance, cooperative behaviour with some reservations.
4. **Frankl 4 (Definitely Positive):** Good rapport with the dentist, interest in the procedure, laughter, and enjoyment during treatment.

**Section F: participants’ preferences for the treatment methods**

- 1. Chemo-mechanical caries removal method
  2. Conventional drilling method

**Section G: Assessment of** **the treatment outcome of participants treated with chemo-mechanical agent and conventional rotary instrument** using **“**Established Criteria for Evaluation of Failures in Different Treatment Procedures and Restorations’’.

Type of restorative material used (GIC or Composite resin).

Code 0 = present, correct.

Code l = present, slight defect at the margin and/or wear of the surface of less than 0.5 mm deep; no repair needed.

Code 2 = present, defect at the margin and/or wear of the surface of 0.5-1.0 mm in depth; repair needed

Code 3 = present, but gross defect at the margin and/or wear of the surface of 1.0 mm or more in depth; repair needed.

Code 4 = not present, restoration has (almost) completely disappeared; treatment needed.

Code 5 = not present, because other treatment has been performed for whatever reason.

Code 6 = tooth not present for whatever reason.

Code 9 = restoration cannot be diagnosed.

Assessed with 0.5 millimetre ball-tip CPI periodontal (WHO) probe.
